# Ifosfamide-induced nephrogenic diabetes insipidus responsive to supraphysiologic doses of intravenous desmopressin 

**DOI:** 10.5414/CNCS110589

**Published:** 2021-07-01

**Authors:** Mohammad A. Sohail, Mohamed Hassanein, Hernan Rincon-Choles

**Affiliations:** 1 Department of Internal Medicine and; 2 Department of Nephrology and Hypertension, Glickman Urological & Kidney Institute, Cleveland Clinic, Cleveland, OH, USA

**Keywords:** ifosfamide, nephrogenic diabetes insipidus, Fanconi syndrome, supraphysiologic desmopressin

## Abstract

Nephrogenic diabetes insipidus (DI) refers to the reduction in the ability of the kidney to concentrate urine, which can be caused by partial or complete resistance at the site of action of anti-diuretic hormone (ADH) in the collecting tubules. Ifosfamide-induced nephrogenic DI typically occurs concomitantly in patients who have other signs of tubular toxicity consistent with Fanconi syndrome including glucosuria, aminoaciduria, and hypophosphatemia. We present a case of a 36-year-old female with recurrent synovial cell sarcoma of the pleural membranes, treated with ifosfamide-based chemotherapy, who was admitted to the hospital for the management of polyuria, hypotension, as well as electrolyte derangements including hypokalemia, hypophosphatemia and non-anion gap metabolic acidosis, 1 week after receiving a cumulative ifosfamide dose of 7.5 g/m^2^. Nephrogenic DI was indicated by polyuria as well as a urine osmolality to plasma osmolality ratio of less than 1.5 following a trial of intravenous desmopressin, but the patient’s acute kidney injury on presentation precluded the early employment of thiazides and non-steroidal anti-inflammatory drugs (NSAIDs). Instead, the patient’s polyuria and urine osmolality improved only after the administration of repetitive supraphysiologic doses of intravenous desmopressin. Our case reiterates that patients with non-hereditary nephrogenic DI may have partial rather than complete resistance to ADH and highlights that desmopressin may be considered in patients with ifosfamide-induced nephrogenic DI to prevent severe volume depletion, especially in patients who have persistent symptomatic polyuria despite maintaining a careful fluid balance and pharmacological therapy with NSAIDs and diuretics, or if the patient’s clinical condition precludes the use of these strategies.

## Introduction 

Diabetes insipidus (DI) represents a group of hereditary and acquired disorders of water homeostasis, which involves the excretion of large volumes of dilute urine, typically greater than 3 L every 24 hours with a urine osmolality of less than 300 mOsm/kg [1]. Nephrogenic DI refers to the reduction in the ability of the kidney to concentrate urine, which can be caused by partial or complete resistance at the site of action of anti-diuretic hormone (ADH) in the collecting tubules [2]. Inherited nephrogenic DI can be caused by loss-of-function mutations in the arginine vasopressin receptor 2 (AVPR2) and aquaporin-2 (AQP2) genes since the AVPR2 and AQP2 proteins help concentrate the urine in response to ADH secretion [3]. Causes of acquired nephrogenic DI include infiltrative disorders such as sarcoidosis, electrolyte abnormalities including hypercalcemia and hypokalemia as well as drugs such as lithium [2]. 

On the other hand, central or neurogenic DI is characterized by the decreased release of ADH secondary to damage to the hypothalamus or pituitary gland by tumors, infiltrative diseases as well as surgery [4]. In addition to maintaining a careful fluid balance, thiazide diuretics and non-steroidal anti-inflammatory drugs (NSAIDs) currently constitute the only effective therapy for nephrogenic DI with ADH resistance [5]. Conversely, the control of polyuria in central DI can be achieved by hormone replacement with the ADH analog, desmopressin, since the primary problem in central DI is the deficient secretion of ADH [5]. Nephrogenic DI is a rare manifestation of ifosfamide-induced nephrotoxicity with only four cases reported previously in adults (Table 1). We present a case of ifosfamide-induced nephrogenic DI with subsequent improvement of polyuria only after the administration of repetitive supraphysiologic doses of intravenous desmopressin. 

## Case presentation 

A 36-year-old female, with a past medical history significant for recurrent synovial cell sarcoma of the left pleural membrane 6 months after undergoing a pneumonectomy, received 6 cycles of chemotherapy with doxorubicin and ifosfamide. Eight days following her 6^th^ and final cycle of chemotherapy with a cumulative ifosfamide dose of 50 g/m^2^, she was hospitalized for the management of pancytopenia, neutropenic fever, acute kidney injury, metabolic acidosis, transient Fanconi syndrome, and doxorubicin-induced non-ischemic dilated cardiomyopathy. 

One year later, she developed another recurrence of her primary sarcoma of the left pleura along with metastases to the right pleura and was subsequently restarted on ifosfamide-based chemotherapy. She presented to the hospital 7 days after receiving her 2^nd^ cycle of chemotherapy (cumulative ifosfamide dose of 7.5 g/m^2^), for the management of hypotension, pancytopenia as well as electrolyte derangements including hypokalemia, hypophosphatemia, and non-anion gap metabolic acidosis. Her medication regimen prior to admission also consisted of ondansetron, morphine, omeprazole, senna, and metaxalone. Her blood pressure on admission was 100/69 mmHg, heart rate was 94/min with a regular rhythm, temperature was 36.9 °C, and respiratory rate was 18 with an oxygen saturation of 99% on room air. Pertinent physical examination findings included dry oral mucous membranes, decreased jugular venous pressure, scaphoid abdomen, and reduced skin turgor. Her hospital course was also notable for significant polyuria and worsening of hypotension despite judicious intravenous fluid replacement. 

Laboratory studies on hospital admission (Table 2) were significant for serum sodium 135 mmol/L (normal, 132 – 148 mmol/L), potassium 3.0 mmol/L (normal, 3.5 – 5.0 mmol/L), chloride 113 mmol/L (normal, 98 – 110 mmol/L), bicarbonate 10 mmol/L (normal, 23 – 32 mmol/L), creatinine 1.20 mg/dL, (normal, 0.58 – 0.96 mg/dL; baseline creatinine, 0.5 mg/dL), glucose 75 mg/dL, (normal, 65 – 100 mg/dL), phosphorus 1.8 mg/dL, (normal, 2.5 – 4.5 mg/dL), and uric acid 1.5 mg/dL, (normal, 2.0 – 7.0 mg/dL). Other laboratory studies were significant for a serum osmolality of 283 mOsm/kg H_2_O, (normal, 275 – 300 mOsm/kg H_2_O), urine osmolality of 126 mOsm/Kg H_2_O, (normal, 40 – 1,400 mOsm/kg H_2_O), and a daily urine output of 7 – 8 L. Urinalysis revealed proteinuria (30 to 100 mg/dL) and glucosuria (> 500 mg/dL). Venous blood gas analysis revealed a pH of 7.25 (normal, 7.32 – 7.42), bicarbonate of 13 (normal 23 – 32 mmol/L), and a venous pCO_2_ of 38 mmHg (normal, 42 – 55 mmHg). 

The above lab parameters including hypokalemia, hypouricemia, hypophosphatemia, normal anion gap hyperchloremic metabolic acidosis, and normoglycemic glucosuria were suggestive of Fanconi syndrome, whilst nephrogenic DI was indicated by polyuria and urine osmolality to plasma osmolality ratio of less than 1.5 following a trial of intravenous desmopressin. The patient’s acute kidney injury on presentation precluded the early introduction of thiazides and NSAIDs to treat her nephrogenic DI. Given the fact that most patients with a non-hereditary etiology for nephrogenic DI have partial, as opposed to complete resistance to ADH and that supraphysiologic doses of desmopressin have been demonstrated to increase urine osmolality in patients with nephrogenic DI [6, 7, 8], it was ultimately decided to try to achieve supraphysiologic levels of desmopressin to treat the patient’s nephrogenic DI, especially since there were limitations in implementing the typical treatment regimen of thiazides and NSAIDs. 

Intravenous desmopressin at a dosage of 40 µg every 6 hours did not produce any significant reduction in the urine volume. Her persistent symptomatic polyuria only improved following a trial of intravenous desmopressin at supraphysiologic doses of 80 µg every 6 – 8 hours. Upon improvement of her kidney function, she was started on hydrochlorothiazide 25 mg daily and indomethacin 25 mg 3 times daily, which had to be discontinued later in the clinical course due to her tenuous kidney function. 

**Figure 1:**
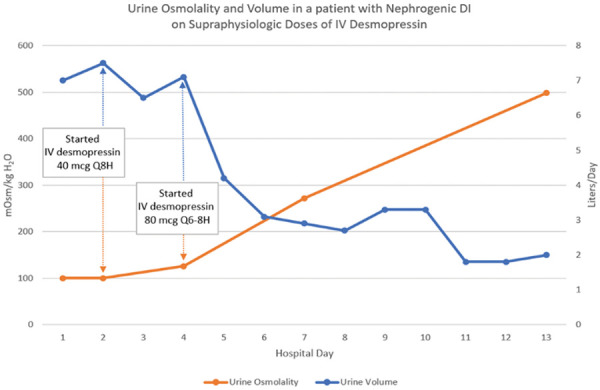
Urine osmolality and volume in a patient with nephrogenic diabetes insipidus on supraphysiologic doses of IV desmopressin. IV desmopressin 40 µg was started on hospital day 2 and IV desmopressin 80 µg was started on hospital day 4.

Throughout the patient’s hospitalization, administration of repeated doses of intravenous desmopressin at supraphysiologic hormone levels led to a steady increase in urine osmolality up to 498 mOsm/kg and a decrease in urine volume to 2 – 3 L a day (Figure 1). Her clinical condition improved, and she was discharged on oral electrolyte supplementation with sodium bicarbonate, phosphorus, and potassium chloride along with oral desmopressin at 0.6 mg twice daily. Her desmopressin was tapered off with complete resolution of her nephrogenic DI within 2 months. 

## Discussion 

Ifosfamide-induced nephrogenic DI typically occurs concomitantly in patients who have other signs of tubular toxicity consistent with Fanconi syndrome, such as glucosuria, aminoaciduria, and hypophosphatemia [9]. This clinical phenomenon was initially reported in 1972 [10], and even though the mechanism for the development of Fanconi syndrome with ifosfamide has since been attributed to the nephrotoxicity of its metabolite, chloroacetaldehyde, which inhibits the NA+/K+-ATPase and consequently, the process of endocytosis in the proximal tubule, it remains unclear how ifosfamide impairs the concentration of urine in the distal tubule to cause nephrogenic DI [11, 12]. 

The most significant predisposing factors that have been reported include exposure to high cumulative doses of ifosfamide of greater than 40 – 60 g/m^2^ [13], prior or concomitant cisplatin administration [14], and patients younger than 16 years of age [15]. In contrast to prior case reports of ifosfamide-induced nephrogenic DI and Fanconi syndrome [16, 17], our patient did not have any of the aforementioned pre-existent risk factors for ifosfamide nephrotoxicity since the cumulative dosage of ifosfamide was only 7.5 g/m^2^. Interestingly, she had previously received a cumulative dose of 50 g/m^2^ of ifosfamide a year prior to her current presentation and had similarly developed transient nephrotoxicity with acute kidney injury, anion-gap metabolic acidosis, and Fanconi syndrome, but without nephrogenic DI. The risk of nephrotoxicity is considered to be low at cumulative ifosfamide doses of 60 g/m^2^ or less [18, 19] which suggests that the patient may be predisposed to developing acute tubular dysfunction with ifosfamide due to unknown risk factors. 

Nephrogenic DI results from the partial or complete resistance of the kidney to the effects of ADH. Consequently, patients with this disorder are considered unlikely to have a good response to hormone administration with desmopressin or to drugs that increase either the kidney’s response to ADH or ADH secretion. Instead, diuretics in combination with NSAIDs can pharmacologically reduce polyuria in patients with nephrogenic DI [20, 21]. Diuretics can induce mild volume depletion to enhance water and sodium reabsorption in the proximal tubule to reduce urine output [20], whilst NSAIDs can increase the kidney’s concentrating ability by inhibiting the synthesis of prostaglandins, which are known to counteract the action of ADH [21]. 

However, our patient’s clinical course highlights a previously unrecognized therapeutic aspect in the management of ifosfamide-induced nephrotoxicity; that is, the response of the persistent symptomatic polyuria caused by nephrogenic DI to repetitive supraphysiologic doses of intravenous desmopressin, particularly in patients with acute kidney dysfunction precluding the use of NSAIDs and thiazides. Administering supraphysiologic levels of desmopressin to our patient may have increased the effects of desmopressin on the kidney to a clinically significant degree since most patients with non-hereditary nephrogenic DI have partial rather than complete resistance to ADH [6, 7, 8]. Desmopressin has been found to increase the urine osmolality by 40 – 45% in patients with nephrogenic DI, an effect that would be expected to produce a corresponding decrease in urine volume [6, 7, 8]. Our patient’s desmopressin responsiveness may also suggest a partial down-regulation of the basolateral AVPR2, which is also the mechanism for partial nephrogenic DI observed in patients with AVPR2 gene mutations [22]. In addition, desmopressin was tapered off as her DI recovered, which may have been secondary to cell recovery after cessation of ifosfamide administration. 

Ifosfamide-induced nephrogenic DI has been rarely described in the literature with only four cases reported in adults to the best of our knowledge (Table 1). All patients were found to have concomitant Fanconi syndrome and were exposed to cumulative doses of ifosfamide ranging from 7.5 to 44 g/m^2^. Management of nephrogenic DI in these reported cases mostly involved administration of hypotonic intravenous fluids, correction of electrolyte abnormalities as well as treatment with diuretics such as amiloride [16, 17, 23]. However, there was one instance of a 26-year-old female with right pterygoid fossa rhabdomyosarcoma who developed ifosfamide-induced nephrogenic DI whose symptoms of polyuria and polydipsia improved with intranasal desmopressin at a supraphysiologic dosage regimen of 20 µg 4 times a day [24]. Furthermore, according to one instance of a patient with lithium-induced nephrogenic DI, the clinical effects of desmopressin can also be enhanced by the concomitant administration of an NSAID [25]. However, in our case, the use of NSAIDs and diuretics during the clinical course was very limited due to the patient’s tenuous kidney function. 

In summary, we report a case of ifosfamide-induced nephrogenic DI that responded to supraphysiological doses of intravenous desmopressin. Our case highlights that desmopressin may be considered in patients with ifosfamide-induced nephrogenic DI to prevent severe volume depletion, especially in patients who have persistent symptomatic polyuria despite maintaining a careful fluid balance and pharmacological therapy with NSAIDs and diuretics, or if the patient’s clinical condition precludes the use of the aforementioned strategies. 

## Funding 

The authors received no financial support for the research, authorship, and/or publication of this article. 

## Conflict of interest 

The authors declare that there are no conflict of interest. 

**Table 1.** Clinical characteristics and treatment of patients with ifosfamide-induced nephrogenic diabetes insipidus.

Case report	Age/Sex	Cumulative dose of ifosfamide (g/m^2^)	Presence of Fanconi syndrome	Employed therapy for diabetes insipidus	Reported clinical outcomes
Negro et al. (1998) [16]	48/Female	41	Yes	Intravenous fluids	Serum electrolytes and kidney function normalized in 20 days
Ingemi et al. (2012) [17]	54/Male	44	Yes	Intravenous fluids and amiloride 20 mg 1 – 2 times/day	Resolution of hypernatremia in 5 days; Impaired kidney function one month after discharge
Kamran et al. (2013) [24]	28/Female	23.4	Yes	Intranasal desmopressin 20 µg 4 times/day	Nephrogenic DI resolved within 2 weeks
Leem et al. (2014) [23]	61/Male	7.5	Yes	Intravenous fluids	The patient died of uncorrected metabolic acidosis 9 days after chemotherapy
Our Case	36/Female	7.5	Yes	Intravenous desmopressin 80 µg 3 – 4 times/day	Urine osmolality increased to 498 mOsm/kg and urine volume decreased to 2 – 3 L/day, nephrogenic DI resolved within 2 months

DI = diabetes insipidus.

**Table 2.** Laboratory serum and urine results obtained 7 days after the start of the 2nd cycle of ifosfamide.

Laboratory test parameter	Baseline values	Day 7 after ifosfamide	Reference range
Serum			
Sodium (mmol/L)	140	135	132 – 148
Potassium (mmol/L)	4.2	3.0	3.5 – 5.0
Bicarbonate (mmol/L)	25	10	23 – 32
Chloride (mmol/L)	105	113	98 – 110
Blood urea nitrogen (mg/dL)	9	10	7 – 21
Creatinine (mg/dL)	0.5	1.20	0.58 – 0.96
Anion Gap (mmol/L)	10	12	9 – 18
Magnesium (mg/dL)	2.0	1.1	1.7 – 2.3
Inorganic phosphorus (mg/dL)	2.8	1.8	2.5 – 4.5
Calcium (mg/dL)	8.5	9.1	8.6 – 10.0
Albumin (g/L)	4.0	3.9	3.9 – 4.9
Osmolality (mOsm/kg)	275	283	275 – 300
Glucose (mg/dL)	95	75	65 – 100
Uric acid (mg/dL)	2.7	1.5	2.0 – 7.0
Urine			
Volume (mL/24h)	2,780	7,470	800 – 2,000
Osmolality (mOsm/kg)	–	126	40 – 1,400
Specific gravity	1.020	1.007	1.005 – 1.030
Glucose (mg/dL)	Negative	500	Negative
Creatinine (g/24h)	–	1.614	0.8 – 1.8
Phosphorus random (mg/dL)	–	151.2	7 – 140
Sodium (mmol/24h)	–	239	40 – 220
Potassium (mmol/24h)	–	178	30 – 99
Protein (mg/dL)	Negative	30 – 100	Negative
